# QTL analysis for chalkiness of rice and fine mapping of a candidate gene for *qACE9*

**DOI:** 10.1186/s12284-016-0114-5

**Published:** 2016-08-22

**Authors:** Yang Gao, Chaolei Liu, Yuanyuan Li, Anpeng Zhang, Guojun Dong, Lihong Xie, Bin Zhang, Banpu Ruan, Kai Hong, Dawei Xue, Dali Zeng, Longbiao Guo, Qian Qian, Zhenyu Gao

**Affiliations:** 1 State Key Laboratory of Rice Biology, China National Rice Research Institute, Hangzhou, 310006 China; 2 College of Life and Environmental Sciences, Hangzhou Normal University, Hangzhou, 310036 China

**Keywords:** Chalkiness, Area of chalky endosperm, QTL analysis, Fine mapping, Rice

## Abstract

**Background:**

An ideal appearance is of commercial value for rice varieties. Chalkiness is one of the most important appearance quality indicators. Therefore, clarification of the heredity of chalkiness and its molecular mechanisms will contribute to reduction of rice chalkiness. Although a number of QTLs related to chalkiness were mapped, few of them have been cloned so far.

**Results:**

In this study, using recombinant inbred lines (RILs) of PA64s and 9311, we identified 19 QTLs associated with chalkiness on chromosomes 1, 4, 6, 7, 9 and 12, which accounted for 5.1 to 30.6 % of phenotypic variations. A novel major QTL *qACE9* for the area of chalky endosperm (ACE) was detected in Hainan and Hangzhou, both mapped in the overlapping region on chromosome 9. It was further fine mapped to an interval of 22 kb between two insertion-deletion (InDel) markers IND9-4 and IND9-5 using a BC_4_F_2_ population. Gene prediction analysis identified five putative genes, among which only one gene (*OsAPS1*), whose product involved in starch synthesis, was detected two nucleotide substitutions causing amino acid change between the parents. Significant difference was found in apparent amylose content (AAC) between NIL*qACE9* and 9311. And starch granules were round and loosely packed in NIL*qACE9* compared with 9311 by scanning electron microscopy (SEM) analysis.

**Conclusions:**

*OsAPS1* was selected as a novel candidate gene for fine-mapped *qACE9*. The candidate gene not only plays a critical role during starch synthesis in endosperm, but also determines the area of chalky endosperm in rice. Further cloning of the QTL will facilitate the improvement of quality in hybrid rice.

## Background

Rice is one of the most important food crops, and fed more than half of the population in the world. In recent years, with the increase of living standard, more and more attention has been paid on rice quality, including the appearance quality, processing quality, nutritional quality, and cooking and eating quality, etc. Chalkiness is an important indicator of the appearance quality for rice. As the opaque part in endosperm, it is an optical character caused by the air-gap of loose arrangement between proteinoplast and amyloplast. Chalkiness can be evaluated by indexes, including area of chalky endosperm (ACE), degree of chalky endosperm (DCE) and percentage of grains with chalkiness (PGWC) when it is associated with high level of damage to the kernel during milling, and thus to a reduction in head rice recovery (Del Rosario et al. 1968). Furthermore, when chalky grain is steamed or boiled, cracks develop readily, reducing the palatability of the cooked product (Nagato and Ebata 1959; Cheng et al. 2005). Therefore, clarification of the heredity of chalkiness and its molecular mechanisms is of important significance to reduce the chalkiness, improve the appearance quality and the commercial value of rice (Tan et al. 2000).

In past decade, several rice mutants associated with chalkiness have been identified and a few genes have been cloned. The *OsPPDKB* gene, which control carbon flow into starch and lipid biosynthesis during grain filling and *starch synthase IIIa* (*SSIIIa*), whose product plays an important role in the elongation of amylopectin chains were cloned by Kang et al. (2005) and Fujita et al. (2007) subsequently. The abnormal growth and loose structure of starch grain in *gif1* mutant caused a significant rise in chalkiness, and the corresponding gene, *GIF1* was fine-mapped on chromosome 4, which encode a cell-wall invertase required for carbon partitioning during early grain filling (Wang et al. 2008). With map-based cloning strategy, She et al. (2010) identified *FLO2* gene on chromosome 4, whose product participated in production of storage starch and storage proteins in the endosperm.

Differences between cultivars in their responsiveness of *FLO2* expression during high-temperature stress indicated that *FLO2* may also be involved in heat tolerance during seed development. However, as a typical quantitative trait, chalkiness is vulnerable to environmental conditions, especially the temperature in the filling stage, when starch is accumulated in endosperm (Lanning et al. 2011; Siebenmorgen et al. 2013). To elucidate the effect of high temperature on grain-filling metabolism, Yamakawa et al. (2007) exposed caryopses of high temperature-tolerant and sensitive cultivars to high temperature (33 °C/28 °C) or control temperature (25 °C/20 °C) during the filling stage, and found that the starch synthesis-related genes, for example, *GBSSI*, were down-regulated at transcript level by high temperature, whereas those for starch-consuming α-amylases and heat shock proteins were up-regulated. In general, high temperature resulted in the occurrence of grains with various degrees of chalky appearance. Nevertheless, there were some varieties not influenced by the high temperature. Murata et al. (2014) developed an *Apq1*-NIL to evaluate the effect of temperature on various agronomic traits, and found that there is no significant difference in percentage of perfect grains (PPG) of the *Apq1*-NIL under high temperature and normal conditions, although PPG of the parent ‘Koshihikari’ is lower under high temperature.

The identification of quantitative trait loci (QTLs) for rice chalkiness and elucidation of the underlying genetic regulation mechanism are necessary for the development of markers for marker assisted selection (MAS) strategies in rice breeding (Yan and Bao 2014). Series of QTLs related to chalkiness have been mapped hitherto by different populations including DH, F_2_ and RIL. Zeng et al. (2002) detected 9 QTLs for chalkiness on chromosomes 8, 11 and 12 respectively with 127 DH lines from transverse section, flank section and belly section. And the intervals and peaks of QTLs on 3 chromosomes were overlapped, some of them even uniform. A total of 22 QTLs for chalkiness were indentified with a population involving 66 chromosomal segment substitution lines (CSSLs) across eight environments, and 9 QTLs were consistently detected across 8 environments, which indicated the 9 QTL alleles were more stable than other 13 QTL alleles (Wan et al. 2005). By using an F_8_ recombinant inbred line (RIL) population consisting of 261 lines derived from a cross between Koshihikari and C602, Liu et al. (2012) detected three QTLs related to PGWC on chromosomes 5, 8 and 10. Zhao et al. (2015b) used the QK model to declare the usefulness of the targeted genes/QTLs. And *SSIIa* was the major gene for chalkiness and explained up to 17 and 21 % of variation of DEC and PGWC, respectively. In addition, the markers RMw513 and RM18068 were associated with DEC in 6 environments as well, and allelic combinations between *SSIIa*, RMw513 revealed more variations in DEC. Besides, many QTLs were fine mapped in limited region. Guo et al. (2011) narrowed down the *qPGWC-8* to a 142 kb region between two Indel markers 8G-7 and 8G-9. Recently, Sun et al. (2015) identified 10 common QTLs for the percentage of grain chalkiness and the degree of chalky endosperm using high-through-put single nucleotide polymorphism (SNP) genotyping of a CSSLs population, and validated the isoamylase gene (*ISA1*) residing on the *qPGC8-2* region, which preferentially expressed in the endosperm and revealed some nucleotide polymorphisms between the parents. Because chalkiness is controlled by multiple genes and its genetic mechanism is relatively complex, so far, only one major QTL for chalkiness, *Chalk5,* has been cloned in rice (Li et al. 2014).

In this study, the relationship between three indexes for rice chalkiness, ACE, DCE and PGWC were analyzed. Nineteen QTLs for chalkiness were identified using a RIL population derived from the cross PA64s × 9311 based on the high-density SNP based genetic map (Gao et al. 2013). A novel major QTL for chalkiness was fine mapped and one candidate gene was selected, which promote further cloning of the QTL and improvement of quality in hybrid rice.

## Results

### Phenotypic variation of the parents and RILs

**Fig. 1:**
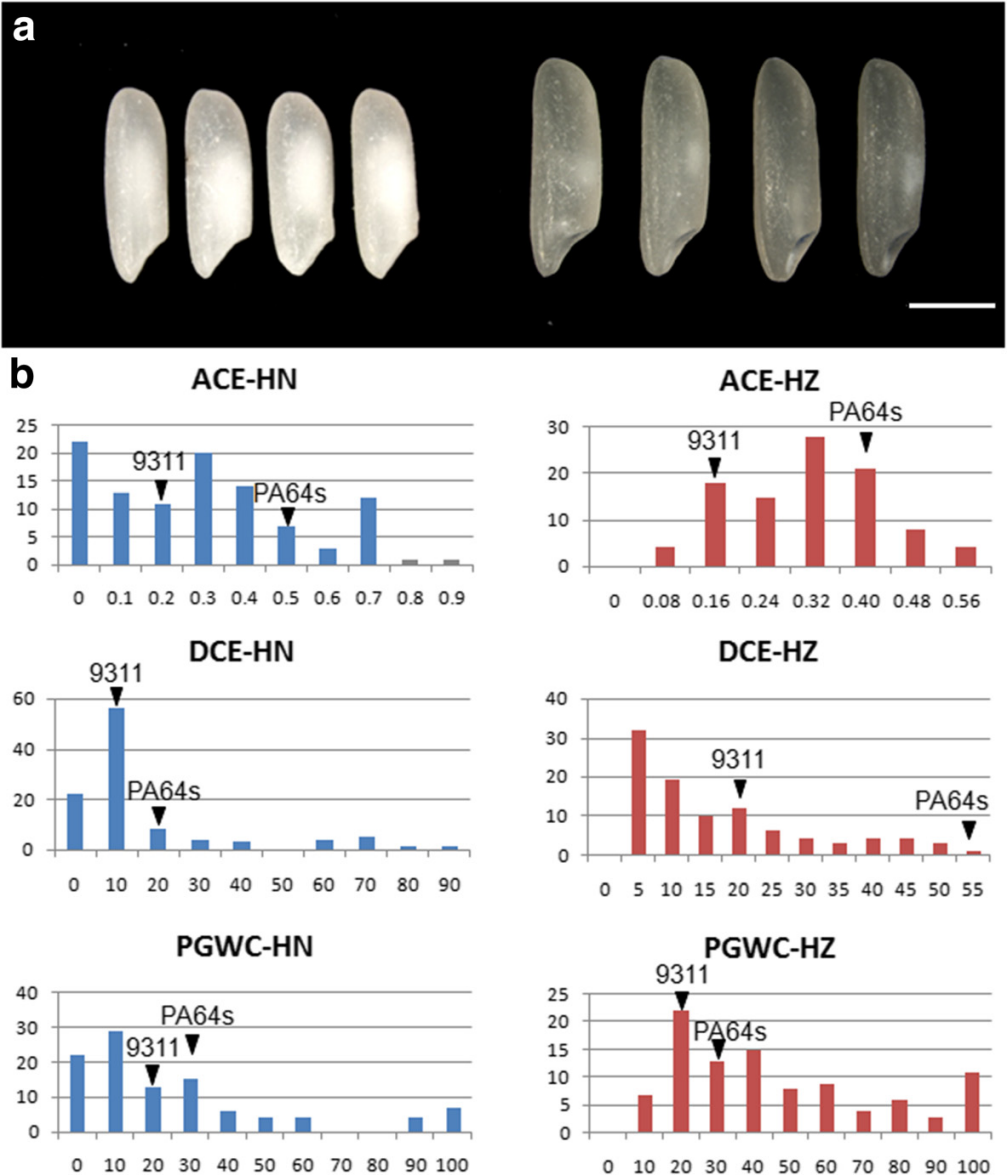
Comparison of chalkiness between two parents and distribution of ACE, DCE and PGWC in the RIL population. **a** Seeds of PA64s (left) and 9311 (right). Bar =2.5 mm. **b** HN represents Hainan and HZ represents Hangzhou

The phenotypic differences between 9311 and PA64s are displayed and summarized in Fig. 1a and Table 1. The basic statistics for the RIL population are also shown in Table 1. Nearly normal distributions and bimodal distributions were observed in the RIL population for ACE, DEC and PGWC in Hangzhou and Hainan, respectively (Fig. 1b), indicating the three traits were controlled by multi-genes in Hangzhou and one or two major genes in Hainan.

**Table 1:** Variations of phenotypes between parents and among RIL in Hainan and Hangzhou

Site	Variety/Population	ACE (cm^2^)	DCE (%)	PGWC (%)
Hainan	9311	0.18±0.03	2.8±0.6	16.0±2.8
	PA64s	0.44±0.04 ^b^	11.7±0.8 ^b^	27.0±4.2 ^a^
	RIL	0.25±0.22	11.5±20.2	22.3±28.8
Hangzhou	9311	0.13±0.03	19.0±5.0	20.0±4.3
	PA64s	0.36±0.03 ^b^	54.0±8.0 ^b^	30.0±3.2 ^a^
	RIL	0.27±0.11	14.4±13.7	42.6±28.1

Mean ± SD (*n* = 6 for parents and *n* = 104 for RIL)

^a^ and ^b^ indicate the least significant difference at 0.05 and 0.01 probability level compared with 9311 in Hainan or Hangzhou, respectively

### Correlation analysis of three chalkiness related traits, heading date and grain shape

The correlations among the three chalkiness characteristics, ACE, DCE and PGWC, heading date (HD) and grain shape traits, such as grain length (GL) and grain width (GW) are shown in Table 2. We identified significantly positive correlations in any couple of ACE, DCE and PGWC in both Hainan and Hangzhou. Meanwhile, the correlations between GW and PGWC, GW and DCE in Hainan and Hangzhou were positive at 5 % and 1 % significant level, respectively.

**Table 2:** Correlation coefficients between ACE, DCE, PGWC and GL, GW, HD

Traits in Hainan	ACE	DCE	PGWC
DCE	0.185 ^a^		
PGWC	0.218 ^b^	0.980 ^b^	
GL	−0.101	0.055	0.071
GW	−0.155	0.224 ^a^	0.204 ^a^
HD	−0.222 ^a^	−0.047	−0.085
Traits in Hangzhou	ACE	DCE	PGWC
DCE	0.428 ^b^		
PGWC	0.460 ^b^	0.984 ^b^	
GL	−0.130	−0.002	−0.019
GW	0.188	0.298 ^b^	0.301 ^b^
HD	−0.035	−0.027	0.002

^a^ and ^b^ indicate at 5 % and 1 % significant level, respectively

### Detection of QTLs for ACE, DCE and PGWC

**Fig. 2:**
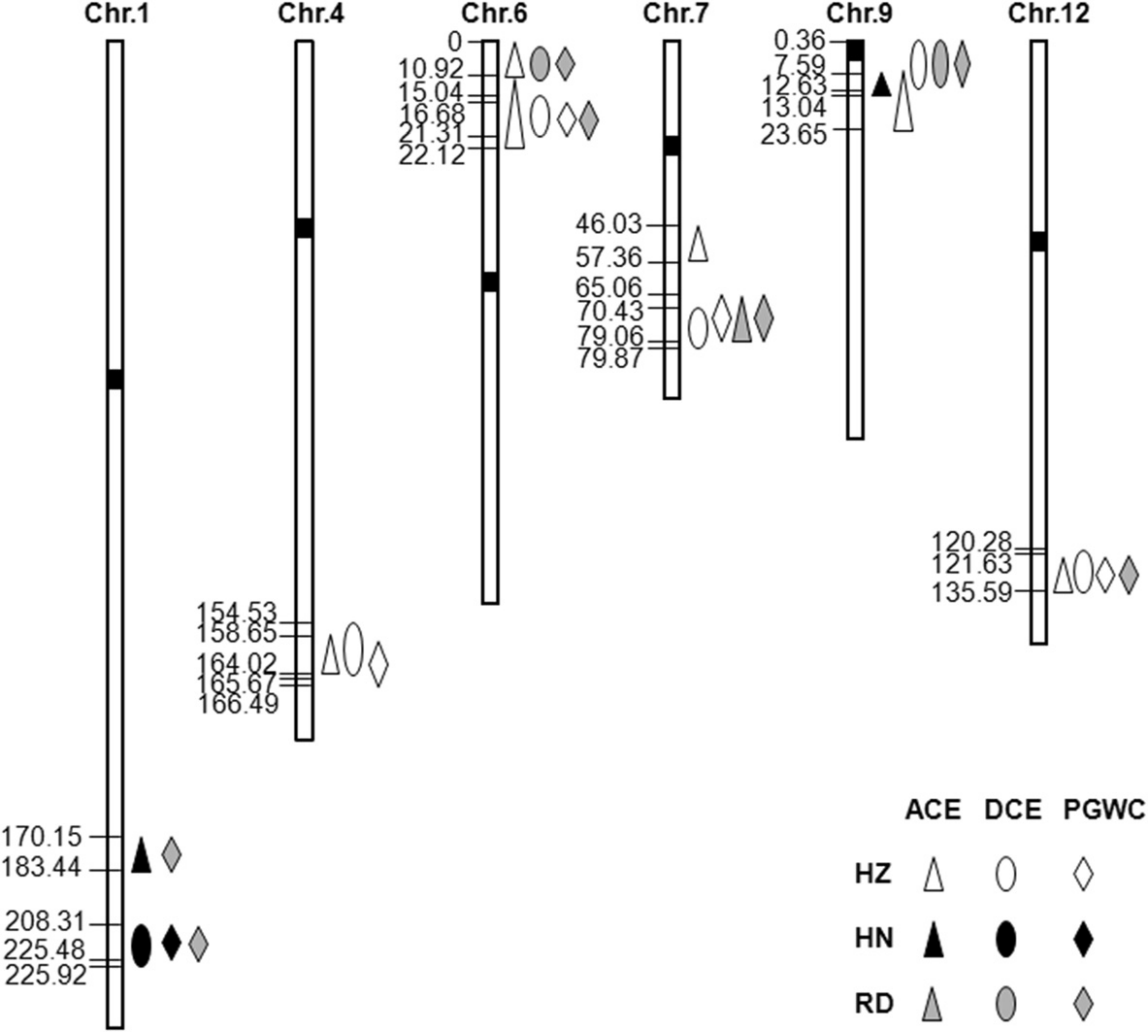
Locations of QTLs on SNP map. Number indicates genetic distance (cM) along each chromosome. HZ represents Hangzhou, HN represents Hainan and RD represents reported QTL

A total of 19 QTLs were detected for the traits of ACE, DCE and PGWC in both Hainan and Hangzhou, distributing on chromosomes 1, 4, 6, 7, 9 and 12 (Table 3; Fig. 2). Eight QTLs for ACE were identified, including 2 QTLs in Hainan and 6 QTLs in Hangzhou. Meanwhile, we detected *qACE9* separately in Hainan and Hangzhou, which explained 12.6 and 13.6 % of the phenotypic variation and located within 7.59 ~ 23.65 cM on chromosome 9. Six QTLs for DCE were detected and each QTL explained 5.2 ~ 30.6 % of phenotypic variation. For the trait of PGWC, 5 QTLs were detected and each QTL explained 5.1 ~ 30.5 % of phenotypic variation. In Hangzhou, we identified *qACE6-2*, *qDCE6* and *qPGWC6*, explained respectively up to 9.2, 30.6 and 30.5 % of the phenotypic variation and located within 10.92 ~ 22.12 cM on chromosome 6.

**Table 3:** QTLs for ACE, DCE and PGWC detected in RIL population in Hainan and Hangzhou

Trait	QTL	Site	Chr.	LOD	*P* value	Genetic distance (cM)	PEV (%)	Subst. effect	Reported QTL
ACE	*qACE1*	Hainan	1	3.83	0.01	170.15–183.44	14.8	0.17	*qPGWC-1a* (Liu et al. 2011)
									*qPGWC1d* (Zhao et al. 2015a)
	*qACE9*	Hainan	9	3.36	0.01	7.59–13.04	12.2	0.16	
	*qACE4*	Hangzhou	4	2.01	0.03	158.65–164.02	8.5	0.05	
	*qACE6-1*	Hangzhou	6	4.01	0.01	0.00–10.92	16.2	−0.09	*qPGWC-6* (Liu et al. 2011), *qDEC6, qPGWC6* (Zhao et al. 2015a), *qCA6-1 N-*, *qCA6-W+* (Peng et al. 2014)
	*qACE6-2*	Hangzhou	6	3.79	0.03	10.92–22.12	9.2	−0.09	*qCR6-H+* (Peng et al. 2014)
	*qACE7*	Hangzhou	7	2.09	0.03	46.03–57.36	5.4	0.05	
	*qACE9*	Hangzhou	9	4.17	0.01	7.59–23.65	13.6	0.09	
	*qACE12*	Hangzhou	12	2.41	0.01	121.63–135.59	6.8	0.06	*qWCR12-D-* (Peng et al. 2014)
DCE	*qDCE1*	Hainan	1	3.43	0.01	208.31–225.92	11.2	−14.17	*qPGWC.NH-1.2* (Bian et al. 2014)
	*qDCE4*	Hangzhou	4	5.97	0.01	154.53–165.67	12.9	8.61	
	*qDCE6*	Hangzhou	6	11.54	0.01	15.04–21.31	30.6	−13.27	*qCR6-H+* (Peng et al. 2014)
	*qDCE7*	Hangzhou	7	2.72	0.03	70.43–79.87	5.6	−5.66	*qPGWC-7* (Zhou et al. 2009), *qWCA7-D+* (Peng et al. 2014)
	*qDCE9*	Hangzhou	9	2.62	0.01	0.36–12.63	5.2	5.46	*qDEC9*, *qPGWC9a*, *qPGWC9b* (Zhao et al. 2015a)
	*qDCE12*	Hangzhou	12	3.19	0.03	120.28–135.59	6.4	6.07	*qWCR12-D-* (Peng et al. 2014)
PGWC	*qPGWC1*	Hainan	1	9.14	0.03	208.31–225.48	11.5	−39.41	*qPGWC.NH-1.2* (Bian et al. 2014)
	*qPGWC4*	Hangzhou	4	7.19	0.01	158.65–166.49	16.3	24.98	
	*qPGWC6*	Hangzhou	6	11.36	0.01	16.68–21.31	30.5	−34.21	*qCR6-H+* (Peng et al. 2014)
	*qPGWC7*	Hangzhou	7	3.69	0.01	65.06–79.06	7.9	−17.38	*qPGWC-7* (Zhou et al. 2009), *qWCA7-D+* (Peng et al. 2014)
	*qPGWC12*	Hangzhou	12	2.51	0.01	121.63–135.59	5.1	13.92	*qWCR12-D-* (Peng et al. 2014)

Among all the 19 QTLs detected with RILs, 6 QTLs were unreported at present, including *qACE9*. There were 16 QTLs distributed in the overlapping region on six chromosomes. A group of QTLs for all three traits were detected in the overlapping region on chromosome 4, 6, 9 and 12. The QTLs for both DCE and PGWC were located in the overlapping interval on chromosomes 1 and 7.

### Fine mapping of *qACE9*

**Fig. 3:**
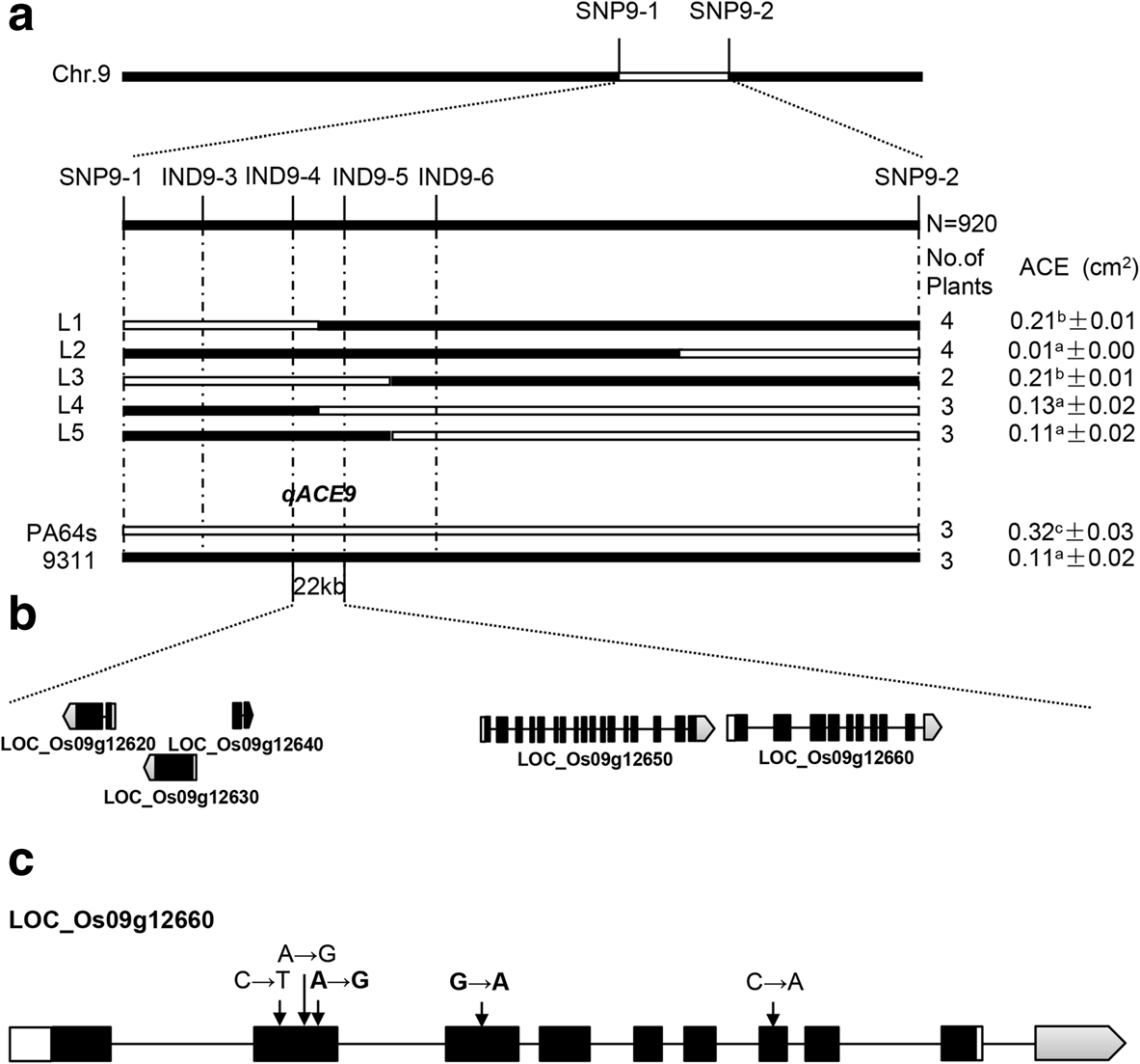
Fine mapping of *qACE9* for ACE. **a** *qACE9* was narrowed down to a 22 kb interval defined by markers IND9-4 and IND9-5. Values represent means ± SD. The superscript letters (a, b and c) indicate significant differences in the trait of the recombinants compared with two parents at the level of 0.01. **b** All the 5 predicted genes in the target region. **c** Structure and mutated sites of the candidate gene. Black boxes represent exons. Bold letter represent the SNPs caused the change of amino acid

**Fig. 4:**
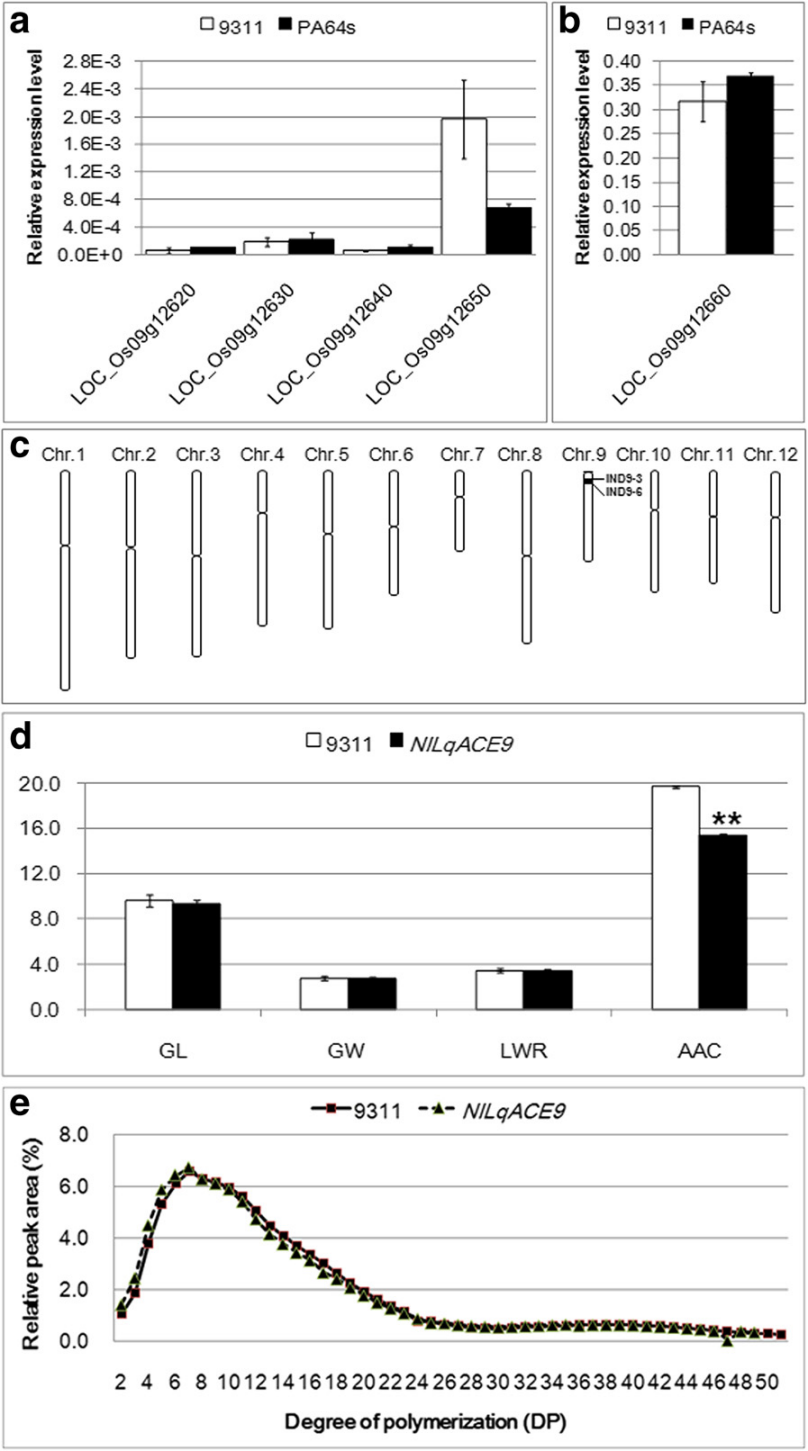
Quantitative real-time RT-PCR analysis of 5 predicted genes in seeds of two parents at filling stage and comparison of grain size (GL, GW and length-width ratio (LWR)), apparent amylose content (AAC) and chain-length distributions of grain amylopectins between NIL*qACE9* and 9311. **a**, **b** Values represent means ± SD of three independent assays. **c** Schematic graph of chromosomes of NIL*qACE9*. **d** Values represent means ± SD of 100 grains for GL, GW and LWR, 3 independent assays for AAC. Unit for Y-axis is cm for GL and GW, and % for AAC. **e** Distribution of chain length distribution of grain amylopectins by FCEP method

For fine mapping of the novel major QTL *qACE9*, a line of RILs with PA64s genotype in the *qACE9* region was selected to backcross with recurrent parent 9311. Then phenotypic character was measured in F_2_ population including 920 individuals derived from a BC_4_F_1_ line with 9311 genetic background exhibiting heterozygous across the entire *qACE9* region screened with markers SNP9-1 and SNP9-2. By comparing the sequences of the parents, four insertion-deletion (InDel) markers were developed. Combining the genotype and phenotype of individuals, the QTL was delimited between two InDel markers IND9-4 and IND9-5 in 22 kb interval (Fig. 3a). The target region contains 5 predicted genes (LOC_Os09g126620, LOC_Os09g126630, LOC_Os09g126640, LOC_Os09g126650 and LOC_Os09g12660) (Fig. 3b) based on Rice Genome Annotation Project Website (http://rice.plantbiology.msu.edu/). Among the 5 predicted genes, there were only 3 predicted genes (LOC_Os09g126620, LOC_Os09g126650 and LOC_Os09g12660) functional annotated. Sequence analysis of the 5 genes in 9311 and PA64s found two synonymous SNPs in LOC_Os09g126650 and 5 SNPs in LOC_Os09g12660 between two parents, among which two SNPs causing amino acid change (Fig. 3c). Therefore, LOC_Os09g12660 were finally selected as the candidate for *qACE9*. A real time PCR was performed for the five genes in the *qACE9* locus (Fig. 4a, b). There were little expression for LOC_Os09g126620, LOC_Os09g126630, LOC_Os09g126640 and LOC_Os09g126650 in two parents, and the expression level for LOC_Os09g12660 was higher in 9311 without significant difference with that in PA64s.

### Characterization of the NIL*qACE9* with 9311 background

**Fig. 5:**
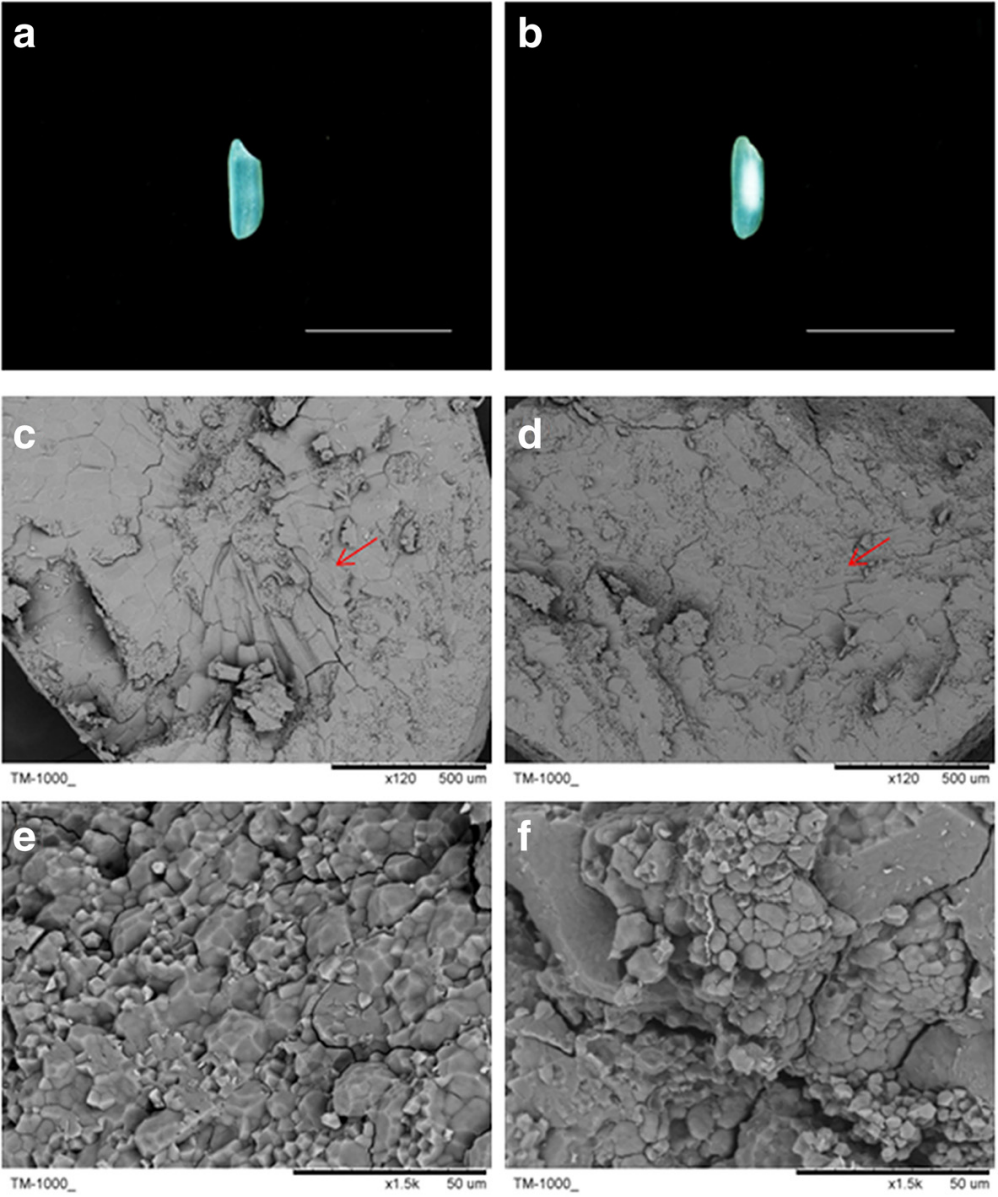
Grain morphology and scanning electron microscopy (SEM) images of starch granule structure. **a**, **c** and **e** from 9311; **b**, **d** and **f** from NIL*qACE9*; The arrowhead in (**c**) and (**d**) represent the position of SEM images in (**e**) and (**f**); Bar represents 1 cm in (**a**) and (**b**)

No significant differences were observed in grain size (GL, GW and length-width ratio (LWR)) between NIL*qACE9*, a NIL carrying homozygous allele of PA64s between InDel markers IND9-3 and IND9-6 (approximately 321.8 kb) with 9311 background, and 9311. However, there was significant difference in apparent amylose content (AAC) between them (Fig. 4d), although no differences observed in amylopectin chain-length distributions (Fig. 4e). NIL*qACE9* had larger area of chalky endosperm, markedly different from that of 9311, though both had similar grain size (Fig. 5a–b). Scanning electron microscopy (SEM) analysis showed the NIL*qACE9* starch granules were round and loosely packed, and very different from polyhedral and densely packed starch granules of 9311 (Fig. 5c–f).

## Discussion

Chalkiness is an important trait of rice appearance. High positive correlations were found between ACE, DCE and PGWC in both Hainan and Hangzhou demonstrated chalkiness related traits were significantly correlated. In addition, GW was found correlated positively with PGWC and DCE in both environments, which consistent with overlapping regions of *qGW4*, *qPGWC4* and *qDCE4* detected in Hangzhou (Gao et al. 2013). This conclusion was also proved by Bian et al. (2013), they use a segregating population derived from sgw (low GW) and cultivar 9311 (high GW) to detect *qsgw7* associated with GW, and developed the NIL*qsgw7* which show lower grain width and chalkiness of brown rice than 9311.

Up to now, many rice mutants associated with chalkiness have been identified and a few genes have been cloned. Although only one QTL for chalkiness was cloned, many QTLs have been reported related to the trait. The *qPGWC-6* and *qPGWC-7* for PGWC located overlapped with our mapped region on chromosomes 6 and 7, and the latter was fine mapped to a 44 kb interval (Zhou et al. 2009). Using a set of CSSLs with ‘Asominori’ genetic background, Liu et al. (2011) detected 6 and 9 QTLs respectively for ACE and PGWC, and two QTLs, *qPGWC-1a* and *qPGWC-6* were also in the same region mapped here. Peng et al. (2014) detected 79 QTLs associated with chalkiness traits using five populations across two environments, among which 5 QTLs in the overlapping region, *qCA6-1 N-*, *qCA6-W+*, *qCR6-H+*, *qWCA7-D+* and *qWCR12-D-* also detected by us. With a population composed of 37 introgression lines (ILs) of Habataki in the background of Sasanishiki, 54 QTLs were identified for grain quality across two different environments (Bian et al. 2014). Among them, the *qPGWC.NH-1.2* for percentage of grains with chalkiness was located in the same region indentified by us on chromosomes 1. Recently, Zhao et al. (2015a) used two sets of RILs derived from reciprocal crosses between Lemont and Teqing to study the genetic basis of chalkiness. A total of 53 and 68 QTLs were detected for DEC and PGWC respectively, among which *qDEC6, qDEC9, qPGWC6, qPGWC1d, qPGWC9a, qPGWC9b* were also identified here in the overlapping region. Because of no difference in CDS of *chalk5* between 9311 and PA64s by sequencing, there was no QTL detected in the region on chromosome 5 here.

In our study, a novel major QTL for ACE, *qACE9* was fine mapped to a 22 kb interval. Comparison of DNA sequence of 5 predicted genes between 9311 and PA64s found two SNPs in one candidate gene LOC_Os09g12660 (*OsAPS1*) caused amino acid changes, that is A_427_ to G_427_ caused Serine to Aspartic, and G_634_ to A_634_ caused Glycine to Asparagine. They both located in the nucleotidyl transferase domain in small subunit of AGPase. There were two small subunits and four large subunits of AGPase, named OsAPS1, OsAPS2, OsAPL1, OsAPL2, OsAPL3 and OsAPL4, which compose one of four classes of enzymes for starch biosynthesis (Tian et al. 2009). Akihiro et al. (2005) cloned all six subunits in Nipponbare. Moreover, comparison of the deduced amino acid sequences of OsAPS1 and OsAPS2 showed high homology between them. Both of the small and large subunits were necessary to ensure the function of AGPase, which was essential for starch synthesis in the seed endosperm. To determine whether the *OsAGPS2* play a critical role during starch synthesis in developing rice endosperm, Lee et al. (2007) isolated mutant *osagps2* for OsAGPS2 by reverse genetic PCR screening of a rice T-DNA insertion library. The levels of AGPase activity and the starch content in *osagps2* were found to be remarkably reduced to 20 and 31 % of the wild type respectively in developing endosperms. Scanning electron microscopy showed that starch granules in the *osagps2* mutants are smaller in size and rounder in shape when compared to those from wild type endosperm. Because of high homology of *OsAPS1* and *OsAPS2* proteins, together with no difference in expression level for *OsAPS1* between the parents, therefore, the difference in chalkiness between 9311 and PA64s may be caused by *OsAPS1* at protein level rather than RNA level.

## Conclusion

In the study, using high-density SNP linkage map, 19 QTLs for rice chalkiness were detected in Hangzhou and Hainan. With the BC_4_F_2_ population derived from a RIL and 9311, *qACE9*, a new major QTL for the area of chalky endosperm (ACE), was fine mapped within 22 kb physical interval on chromosome 9. One candidate gene, *OsAPS1*, whose product reported involved in starch synthesis, was finally selected based on difference in coding sequence causing amino acid change between the parents. There were significant differences in apparent amylose content (AAC) and starch granules structure in endosperm between NIL*qACE9* and 9311. It helps further cloning of the QTL and facilitates the improvement of rice quality.

## Methods

### Development of mapping population

A total of 104 RILs derived from the cross of the *indica* variety 9311 and the light-thermo-sensitive genic male sterile line PA64s were used in this study. The population was developed in the experimental fields at China National Rice Research Institute in Hangzhou, Zhejiang Province, and in Lingshui, Hainan Province, China. To develop a NIL containing the QTL for ACE, *qACE9* detected both in Hainan and Hangzhou on chromosome 9, a line of RILs with PA64s genotype in the *qACE9* region was selected to backcross with recurrent parent 9311. Two markers SNP9-1 and SNP9-2 (Table 4) were used for marker assisted selection (MAS) of each generation. As a result, a BC_4_F_1_ line, with 9311 genetic background exhibiting heterozygous across the entire *qACE9* region, was constructed. After self-crossing, a BC_4_F_2_ population was obtained for fine mapping of *qACE9*. A NIL carrying homozygous allele of PA64s in the target QTL region between InDel markers IND9-3 and IND9-6 (Table 4), designated NIL*qACE9*, was also developed from one chromosome segment substitution line (CSSL) with 93–11 background (Fig. 4c).

**Table 4:** Primers for InDel markers and SNP markers developed

Primer	Forward (5′-3′)	Reverse (5′-3′)	Type
SNP9-1	AGCATAGTTGTAAAACATGCCAGAC	TGCCGGAAAATAAATTCACCC	SNP
SNP9-2	TTCGTATTTTATAGAACAGAGGG	TGTGTGCTAAGAACACAAAGG	SNP
IND9-3	CAGTATATGTGACGGAGCTATTTTC	ATTATCCTTGGTTATACACCG	InDel
IND9-4	CCAACCTCCAAGACTAGATGAAGTT	AACATTACTTGTGGGCTCTTG	InDel
IND9-5	TTTGATCGGACAATTTGTTT	AAAAACCGGAAAAAGAAAAG	InDel
IND9-6	TAGATGGGCCAGTTCAAATTG	ACCATATGTTTTTACATTTGATTGC	InDel

### Measurement of chalkiness related traits

The plot size was four rows of six plants with a 35 × 35 cm spacing. Mature seeds of each line were harvested 30 days after heading and dried in an electro-thermal incubator (ZXDP-A2160, Shanghai) at 30 °C for 72 h after harvest. The dried seeds (20 g) were dehulled and polished, and then intact seeds were selected for measurement of chalkiness related traits. The ACE, DEC and PGWC were evaluated according to He et al. (1999) and National Standard of People Republic of China (NSPRC 1999). To separate chalky grains from vitreous grains, 40 grains selected at random per entry were assessed on a chalkiness scanner. PGWC was calculated based on these photographs. ACE and the area of the whole endosperm for each grain were estimated visually by the software, and the values for both were averaged. DEC was the ratio of ACE to the area of the whole endosperm.

### Scanning electron microscope (SEM) analysis of rice grains

For observation of starch granules, unbroken milled rice was cut transversely with a blade, and the pieces were stuck onto a 12-mm aluminum stub, and sputtered with gold on a polaron sputter coater. Samples were viewed with SEM and diameters of starch granules were estimated on the basis of the scale bar provided on the captured image.

### Measurement of apparent amylose content (AAC) and chain-length distribution of starch

AAC was measured following the procedure of Perez and Juliano (1978) with some modifications. Absorbance of the starch solution was determined at 620 nm using the spectrophotometer. The method determining chain-length distribution of starch was essentially identical to the procedure described by Fujita et al. (2012) using the fluorescence capillary electrophoresis (FCEP) method of O’Shea and Morell (1996) in a P/ACE MDQ Capillary Electrophoresis System (Beckman Coulters, CA, USA).

### Statistical analyses and QTL analysis

All statistical analyses were completed using the SAS (Statistical Analysis System) v8.01. QTL analysis was performed with the MultiQTL package (www.multiqtl.com) using the maximum likelihood interval mapping approach for the RIL-selfing population. For major-effect QTLs, the LOD threshold was obtained based on a permutation test (1000 permutations, *P* = 0.05) for each dataset. QTLs were named according to Mccouch et al. (1997).

### Design of markers for fine mapping

Primers were designed in *qACE9* region on the basis of insertions/deletions (InDels) and SNPs identified between 9311 and PA64s (Table 4) (Gao et al. 2013). Genotypes of SNP markers were screened by high-resolution dissociation curve analysis system (LightScanner 96, Idaho Technology Inc.).

### Real time PCR analysis

Total RNA was isolated from panicles at filling stage with RNA extraction kit (Axygen). DNase treatment, cDNA synthesis, primer design and SYBR Green I real time PCR were carried out as described (Vandesompele et al. 2002) using a Rever Tra Ace® qPCR-RT kit (TOYOBA, Japan). Real time PCR amplification mixtures (10 μl) contained 50 ng template cDNA, 2 × SYBR Green PCR Master Mix (Applied Biosystems), and 200 nM forward and reverse primers. Reactions were conducted on an ABI PRISM_7900HT Sequence Detector (Applied Biosystems). The relative expression level of each transcript was obtained by comparing to the expression of the *Actin* gene. Primers for candidate genes and *Actin* are listed in Table 5.

**Table 5:** Primers for real time PCR analysis

Primer	Forward (5′-3′)	Reverse (5′-3′)	Gene
RT-62	CTGCAGGCGAAGAAGGAT	GTGATCACCGTGTAGTTCGC	LOC_Os09g12620
RT-63	TACTACGCCTCGGTGGAGA	TCCGGGTAGACGTCGAAT	LOC_Os09g12630
RT-64	ACGTGGATTCAGCCAAATG	AATGGCAAGATCTCCGTAGG	LOC_Os09g12640
RT-65	ACATGCGCAAATATGGTTGT	CCAGAGAACACCACACCAAC	LOC_Os09g12650
RT-66	ATTCAGGCCCACAGAGAAAC	TGATCCTCCCTTCATCATCA	LOC_Os09g12660
Actin	CCATTGGTGCTGAGCGTTT	CGCAGCTTCCATTCCTATGAA	LOC_Os03g50885
